# Zoonoses in the Democratic Republic of Congo: anthrax outbreak in the province of Nord-Kivu has prompted a health alert

**DOI:** 10.1097/MS9.0000000000003684

**Published:** 2025-08-01

**Authors:** Joshua Ekouo, Christian Tague, Hermann Yokolo, Lidoine Djom, Dujardin Makeda, Areeba Aamir Ali Basaria, Ismat Fatima, Aymar Akilimali

**Affiliations:** a Department of Research, Medical Research Circle (MedReC), Goma, DR Congo; b Faculty of Medicine, Université libre des pays des grands lacs, Goma, DR Congo; c Department of Medicine, Dow University of Health Sciences, Karachi, Pakistan; d Department of Medicine, Jinnah Sindh Medical University, Karachi, Pakistan

Anthrax is a serious infection (bacterial zoonosis) that mainly affects domestic and wild herbivores. This infection is caused by a spore-forming bacillus called *Bacillus anthracis*, which is able to survive for many years in the environment, from where it can contaminate humans through direct contact with infected animals, their by-products, or contaminated meat. The onset of symptoms varies depending on the mode of infection. They can manifest as a skin lesion with a dark crust or progress to respiratory problems^[1]^. In the Democratic Republic of Congo (DRC), several recent outbreaks have been reported in recent years. Especially in the province of North Kivu and the Virunga National Park, where recent episodes of high mortality in hippopotamuses as well as human cases have been reported^[2]^.

In June 2024, several suspected cases of anthrax were reported in the Bashu chiefdom, located in Beni territory, before recently spreading to Lubero. Since April 11, the local health zone has reported several suspected cases there, including four identified individuals: one unfortunately died, and the other three were transferred to the UCG/Horizon university clinics in Butembo for specialized care. According to the most recent WHO reports, as of 16 May 2025 suspected human cases have been reported in North Kivu Province, with one confirmed case and one death, following the deaths of dozens of buffalo and hippopotamuses in Virunga National Park^[3]^. A total of eight cases have recently been suspected, including five cases from the Lubero health zone and three from Binza^[2,4]^.

The aim of this article is to provide an overview of the anthrax situation in the Democratic Republic of Congo, with a focus on the outbreak in North Kivu. It describes the disease, its modes of transmission, clinical signs, treatment and prevention methods, as well as public health actions taken. The article also offers recommendations to improve the prevention and management of this zoonotic disease in the country. Recent initiatives aimed at standardizing the reporting of artificial intelligence research, such as the TITAN guideline (Transparency In The Reporting of Artificial INtelligence) introduced in 2025, underscore the imperative for methodological rigor, transparency, and reproducibility in machine learning-based studies^[5]^.

According to the most recent data published by the World Health Organization (WHO), 2024 is already seeing a significant increase in anthrax cases. Since the beginning of 2023, 16 human cases have been officially recorded, including three deaths. This figure is particularly worrying given that it represents, in just a few months, an increase in the circulation of *B. anthracis* in certain at-risk areas, particularly in rural and pastoral areas where close contact between humans and livestock favors transmission^[6]^.

Since the beginning of 2024, several other countries in East and Southern Africa have been facing active anthrax epidemics, raising concerns among health authorities and international partners. According to the World Health Organization (WHO), more than 1166 suspected cases have been reported in five countries: Kenya, Malawi, Uganda, Zambia, and Zimbabwe, with 20 deaths recorded to date. Of these cases, 37 have been confirmed by laboratory tests. Zambia, in particular, is facing its worst epidemic since 2011, with 684 suspected cases and four deaths recorded as of 20 November 2024, affecting nine of the country’s ten provinces. Malawi, meanwhile, reported a human case for the first time this year. In Uganda, 13 deaths have been reported, demonstrating the severity of the situation^[4,6]^.

In the Democratic Republic of Congo, recent anthrax outbreaks have been reported in North Kivu (Beni, Lubero) and historically in Virunga, Garamba, and Ituri national parks. The disease affects both humans and animals, with variable human mortality and significant animal losses. *B. anthracis* spores persist for a long time in the soil, favored by specific climatic and environmental conditions. Poverty, chronic insecurity, and population displacement increase the vulnerability of rural communities, often exposed when handling or consuming contaminated animals, thus contributing to the periodic re-emergence of epidemics^[2,5,6]^.

Anthrax represents a significant public health challenge in the DRC, where it intersects with broader issues of food security, conflict, and economic instability. The annual economic impact of anthrax in eastern DRC is estimated at $2.3 million, primarily through livestock losses and healthcare costs. The disease disproportionately affects rural populations, with 85% of cases occurring in agricultural communities. The burden is compounded by limited healthcare infrastructure, with only 34% of health facilities in North Kivu having essential diagnostic capabilities. Globally, anthrax remains a neglected tropical disease affecting predominantly low- and middle-income countries. The WHO estimates 2000–20 000 human cases annually worldwide, with sub-Saharan Africa accounting for 45% of cases. The emergence of antimicrobial resistance in *B. anthracis* strains in several countries raises concerns about treatment effectiveness. The 2001 bioterrorism attacks in the United States highlighted anthrax’s potential as a biological weapon, leading to increased international surveillance and preparedness efforts. Recent outbreaks in West Africa (Ghana-Nigeria, 2023) and East Africa (current multi-country outbreak) demonstrate the regional nature of anthrax transmission and the need for coordinated international response^[1,3,6,7]^.

The ongoing armed conflict in North Kivu presents unique challenges for anthrax prevention and control. Vaccine distribution is severely hampered by insecurity, with over 2.7 million internally displaced persons in the region having limited access to healthcare facilities. Sociocultural factors further complicate control efforts, including traditional practices of bushmeat consumption, particularly during food shortages, and widespread distrust of health authorities following decades of conflict. The collapse of veterinary services in conflict-affected areas has resulted in inadequate livestock vaccination coverage, with reports indicating less than 30% vaccination rates in endemic zones. Additionally, the weak cold chain infrastructure and frequent power outages compromise vaccine efficacy, particularly in rural areas where temperatures can exceed 40°C^[2,4,8,9]^.

Anthrax, also known as anthrax, is transmitted primarily to humans in three main ways: through direct contact with infected animals, through the consumption of improperly prepared contaminated meat, and through exposure to the blood or bodily fluids of an infected person. Secondarily, it can be transmitted through inhalation of spores^[10]^. High-risk groups are primarily butchers, livestock farmers, veterinarians, rural populations, those who consume poorly or uncooked meat, and wildlife workers^[7]^.

The clinical variability of anthrax includes cutaneous, gastrointestinal, and pulmonary forms. Cutaneous anthrax begins as a pruritic papule that progresses to a black eschar, accompanied by edema, lymphadenopathy, and constitutional symptoms such as fever. Complete recovery may require several weeks. Inhaled anthrax begins with a flu-like syndrome, progresses rapidly to respiratory distress, shock, coma, mediastinal lymphadenitis, pulmonary edema, pleural effusion, and can lead to hemorrhagic meningoencephalitis^[11]^. The mortality rate of untreated anthrax varies, reaching nearly 100% for both inhalational and meningeal forms^[12]^. Digestive anthrax varies from asymptomatic to fatal forms, with fever, abdominal pain, hemorrhagic diarrhea, and ascites, and can progress to intestinal necrosis or lethal septicemia^[11]^. The infectious dose of anthrax varies: 10–50 spores cutaneously, 8000–50 000 inhaled, and much higher for the digestive tract. Without treatment, the mortality of anthrax varies from 20% to 60%. Treated, it depends on the form, with high morbidity for inhaled and digestive^[8]^.

As anthrax is a zoonosis most often transmitted by herbivores, including livestock, prevention of this disease most often consists of breaking the cycle of transmission from animals to humans by carefully eliminating the remains of animals that have died from the disease by incineration until thermal sterilization of the underlying soil is achieved, as well as vaccination of animal herds and high-risk individuals, including livestock farmers, military personnel, and laboratory personnel^[9]^. The management of anthrax consists of antibiotic therapy, which is adapted according to the location and severity of the disease. The bacterium *B. anthracis* is sensitive to several antibiotics, including penicillin, aminoglycosides, macrolides, quinolones, carbapenems, tetracyclines, vancomycin, clindamycin, rifampicin, cefazolin, and linezolid^[13]^. The treatment of uncomplicated cutaneous anthrax consists of monotherapy with intramuscular penicillin G or with doxycycline or ciprofloxacin orally. However, when it is complicated (significant edema or cervicofacial involvement), antibiotics must be administered intravenously associated with corticosteroids and dressing of skin lesions. In cases of inhalation anthrax, treatment consists of a combination of a bactericidal agent and a protein synthesis inhibitor, particularly intravenous ciprofloxacin + clindamycin, linezolid, or clarithromycin. In cases of meningitis, triple antibiotic therapy comprising two bactericidal agents from different classes (fluoroquinolone + beta-lactam) + a protein synthesis inhibitor is recommended. For gastrointestinal anthrax, treatment consists of a combination of penicillin G and aminoglycosides (streptomycin or other)^[13,14]^.

## Prevention and control measures

The fight against anthrax in North Kivu province relies on a range of prevention and control strategies aimed at limiting the spread of this zoonosis. First and foremost, routine vaccination of livestock in endemic areas remains a critical measure. It significantly reduces the risk of transmission to humans, particularly in rural communities where contact with animals is frequent. Raising awareness among livestock farmers and local populations through information campaigns is essential to improve hygiene practices, encourage the prompt reporting of suspected cases, and avoid the consumption of meat from animals that have died of unknown causes^[15]^. Furthermore, strengthening epidemiological surveillance and quarantining affected areas allows for rapid detection of outbreaks and targeted intervention. The implementation of a comprehensive One Health approach in North Kivu requires strengthening inter-sectoral collaboration between the Ministry of Public Health, Ministry of Agriculture, and Ministry of Environment. Successful One Health interventions in neighboring countries demonstrate the effectiveness of this approach: in Zambia’s 2023 anthrax outbreak, joint human-animal surveillance systems reduced outbreak duration by 60% compared to previous episodes. In the DRC context, establishing joint investigation teams comprising human and veterinary epidemiologists, implementing shared surveillance platforms, and creating unified rapid response protocols are essential. The approach should include: (1) synchronized vaccination campaigns for both livestock and high-risk humans, (2) joint case investigation protocols, (3) shared laboratory capacity for both human and animal samples, and (4) coordinated risk communication strategies targeting both agricultural and health sectors. Furthermore, environmental monitoring of *B. anthracis* spores in soil and water sources, particularly around burial sites of infected animals, is crucial for long-term prevention^[1,3,7,12]^.

Our analysis does not cover some important aspects. We did not further explore the ecological impact of anthrax on wildlife, nor did we thoroughly assess the sociocultural and economic factors influencing transmission. Specific clinical data for Congolese patients and the practical challenges of providing medical care in rural areas were not explored, which limits the scope of the conclusions and highlights the need for further research on these dimensions.

Molecular characterization of *B. anthracis* strains in the DRC outbreak remains a critical gap in our understanding. Genomic sequencing could provide insights into strain virulence, antibiotic resistance patterns, and transmission dynamics. Recent advances in portable sequencing technologies, such as Oxford Nanopore MinION, offer feasible solutions for resource-limited settings. Phylogenetic analysis of *B. anthracis* isolates from the 2023 Ghana-Nigeria outbreak revealed distinct genetic clusters associated with different transmission routes, informing targeted control strategies. We recommend establishing molecular surveillance capacity through partnerships with regional reference laboratories and implementing standardized protocols for sample collection, storage, and transportation. Spatio-temporal analysis using Geographic Information Systems (GIS) mapping could identify high-risk areas and transmission patterns, guiding resource allocation and intervention prioritization^[3,6,7]^.

Context-specific recommendations for anthrax control in North Kivu include: (1) Establishing mobile vaccination units with armed escort capability to reach conflict-affected areas, targeting a minimum 80% livestock vaccination coverage in endemic zones; (2) Implementing community-based surveillance systems using trained traditional healers and community health workers to identify and report suspicious animal deaths within 24 hours; (3) Developing culturally appropriate risk communication strategies in local languages (Swahili, Nande, Nyanga) addressing traditional practices and building trust with communities; (4) Creating stockpiles of essential antibiotics (ciprofloxacin, doxycycline) in health facilities within 50 km of endemic areas; (5) Establishing rapid diagnostic capacity using point-of-care tests for *B. anthracis* in district hospitals; (6) Implementing secure carcass disposal protocols with community engagement, including compensation mechanisms for farmers who report animal deaths; (7) Strengthening cross-border surveillance with Uganda and Rwanda to prevent transboundary transmission; (8) Developing emergency response protocols for healthcare workers in conflict zones, including personal protective equipment and evacuation procedures^[3,11]^.

## Policy recommendations for stakeholders

To effectively combat anthrax, tailored strategies must be implemented across different stakeholder levels. For healthcare providers, it is essential to establish mandatory training programs focused on anthrax recognition and management, particularly in endemic regions. Standardized clinical protocols should be developed for case management, including clear referral pathways to ensure timely and appropriate care. Enhancing telemedicine capabilities can bridge the gap between rural health facilities and specialist centers, facilitating expert guidance in real time. Additionally, the formation of rapid response teams equipped with appropriate personal protective equipment is critical for immediate intervention during outbreaks.

Policymakers play a vital role in shaping a sustainable and coordinated response. Integrating anthrax prevention into national health security strategies with dedicated budget allocation is necessary for long-term planning. Legal frameworks must be enacted to mandate livestock vaccination in endemic zones, a crucial step in reducing animal-to-human transmission. To encourage early reporting and surveillance, compensation mechanisms should be developed for farmers who report livestock deaths. Furthermore, establishing inter-ministerial coordination mechanisms among health, agriculture, and defense sectors will ensure a unified and efficient response to outbreaks.

At the international level, stakeholders can support capacity building by enhancing laboratory diagnostic capabilities and expanding molecular surveillance. Providing technical assistance for the implementation of One Health approaches will help integrate human, animal, and environmental health strategies. Facilitating cross-border coordination is vital for managing transboundary disease threats, especially in regions with porous borders. Finally, supporting conflict-sensitive health programming, particularly in areas like eastern DRC, will ensure that interventions are effective and context-appropriate^[1,2,10]^.

The resurgence of anthrax in the DRC, particularly in North Kivu, constitutes a major health emergency requiring immediate and sustained intervention. This disease, capable of persisting for long periods in the environment, represents a significant threat due to its potential transmission between animals and humans, high mortality rate without rapid treatment, and the particular vulnerability of communities in conflict-affected areas. The complex interplay of factors including ongoing armed conflict, population displacement, limited healthcare infrastructure, and traditional practices creates a unique challenge requiring innovative solutions. The integrated “One Health” approach, combining human, animal, and environmental health perspectives, coupled with conflict-sensitive programming, is essential for an effective and sustainable response. Immediate priorities include strengthening surveillance systems, implementing context-specific prevention strategies, and building local capacity for outbreak response. Long-term success requires addressing underlying determinants of health, including poverty, food insecurity, and governance challenges. With coordinated action from national authorities, international partners, and local communities, it is possible to reduce the burden of anthrax and build resilience against future outbreaks in the Democratic Republic of Congo.

## Data Availability

Not applicable.
